# Age-related lung structure changes by quantitative assessment: a cross-sectional study in a Chinese male cohort

**DOI:** 10.3389/fragi.2025.1624233

**Published:** 2025-10-23

**Authors:** Meijuan Shi, Youmin Guo, Chenwang Jin, Cong Shen

**Affiliations:** ^1^ Department of Radiology, The Second Affiliated Hospital of Xi’an Jiaotong University, Xi’an, Shaanxi, China; ^2^ Department of PET/CT, The First Affiliated Hospital of Xi’an Jiaotong University, Xi’an, Shaanxi, China

**Keywords:** age-related changes, lung structures, quantitative assessment, LungAge score, lung aging

## Abstract

**Objectives:**

This study aims to investigate age-related alterations of lung structure.

**Methods:**

We retrospectively collected 928 male subjects from an annual lung nodule screening cohort. The quantitative parameters included lung volume (LV), mean lesion density (MLD), emphysema indexes (LAA-910, LAA-910%, LAA-950 and LAA-950%), number of bronchi (NB) and volume of bronchi (VB), as well as ratio of airway to the lung (ALR). The quantitative parameters were calculated for total lung, right lung, left lung, and the individual lobes.

**Results:**

LV and VB peaked in the group of 51–60 years-old and 61–70 years-old, respectively. MLD decreased with age, while LAA-910, LAA-950, LAA-910%, LAA-950%, and ALR all showed an increasing trend with age. LV, NB, and VB of the right lung were larger than those of the left lung, while MLD, LAA-950, LAA-950%, and ALR of the right lung were lower than those of the left lung (*P* < 0.05). The LV of bilateral upper lobes increased with age, while a decline of LV of bilateral lower lobes was observed since the sixties. The MLD of the bilateral lower lobes decreased (*P* < 0.05). The LAA-910%, LAA-950%, and ALR of the 71–80 years-old in all five lobes were higher than those of the other four groups (*P* < 0.05). LAA-950 and LAA-950% of bilateral lower lobes displayed a steeper increase began at 60 years old. We also provide a computational formula, LungAge Score, for the assessment of the structural lung aging features.

**Conclusion:**

Lung aging is not a linear process, and the lung structural alterations in the upper and lower lobes exhibit significant heterogeneity.

## 1 Introduction

Evidence showed that chronic respiratory diseases (CRDs) are the third leading cause of death, responsible for 4.0 million deaths (95% uncertainty interval 3.6–4.3), with a prevalence of 454.6 million cases (417.4–499.1) globally (GBD, 2019 Chronic Respiratory Diseases Collaborators et al., 2023), which imposing a huge burden of death, disability and healthcare costs. Aging is one of the most important risk factors for CRDs, and the worldwide increase in life expectancy has been accompanied by an increase in the prevalence of age-related CRDs (Cho and Stout-Delgado, 2020; Schneider et al., 2021; Skloot, 2017). Thus, the study of age-related lung changes is imperative to preventing or ameliorating CRDs.

The normal aging process of the lungs is associated with structural and functional alterations (Miller, 2010). That means, the “healthy aging” individuals may suffer from age-related disease but retain their functional abilities. Previous studies have shown that the loss of elastic recoil, hyperinflation, air trapping, progressive enlargement of the alveolar ducts and distal alveoli, and an increase in residual volume are the main physiological manifestations in the aging lung (Bowdish, 2019; Kasmani et al., 2023). On one hand, the alterations of lung structure of normal aging may be partly responsible for the increased susceptibility of older persons to lung disorders, such as emphysema-predominant chronic obstructive pulmonary disease, fatal respiratory infection, interstitial lung diseases, and primary lung cancer (MacNee, 2016; Han et al., 2021). On the other hand, these overlaps put forward a challenge in the clinical management of CRDs. Thus, we need to analyze the changes in lung structure in the elderly.

Computer tomography (CT) is a practical and noninvasive method for evaluating lung structures (Jiao et al., 2024). Meanwhile, advanced image post-processing techniques enable the quantitative analysis of changes in lung and branches, which is helpful in understanding the normal aging process. Researchers in our team and other studies have proved the value of the quantitative biomarkers of emphysema, the airway, and air trapping (relative volume change of −860 Hounsfield Unit (HU) to −950 HU) in the clinical management of CRDs (Yin et al., 2019; Jiang et al., 2018; Shen et al., 2025; Shen et al., 2020; Kim et al., 2022; Tanabe et al., 2018). However, there are no studies available for the quantification of age-related changes in the lungs of the CRD-free cohort, which is essential for understanding normal aging.

Thus, we aimed to investigate age-related alterations in lung structure using a quantitative technique to assess lung tissue (lung volume, mean lung density), emphysema (LAA910, LA950), and the bronchial tree (the number of branches and the volume of branches) in a Chinese male cohort from a single institution. Meanwhile, we designed a comprehensive evaluation score, the LungAge score, to assess the physiological aging of lungs.

## 2 Materials and methods

### 2.1 Subjects

This was a post-hoc analysis of a prospective clinical trial, which was registered online (http://www.chictr.org/en/; registration number ChiCTR-OCH-14004934). The Ethics Committee of the Institutional Review Board approved this study (No. 2013-114–1). All participants were fully informed of the nature of the study and provided written informed consent for participation.

In this study, we included only males to avoid factors that contribute to sex-related differences (Terada et al., 2023; Silveyra et al., 2021). Chest CT images of 1,097 subjects were retrospectively collected from August 2017 to December 2019 from an annual lung nodule screening cohort. The inclusion criteria were: 1) male subjects who underwent a non-contrast chest CT scan; 2) age between 18 and 80 years old. The exclusion criteria were: 1) subjects with a congenital deformity of the spine or thorax (n = 25); 2) subjects who had a history of lung illness or any respiratory symptoms (n = 10); 3) subjects with a history of lobectomy or pneumonectomy (n = 10); 4) subjects with noticeable respiratory or motion artifact (n = 37); 5) subjects with apparent lesions in the CT scan, such as the diffused emphysema, bronchiectasis, interstitial lung abnormality, lobar consolidation, nodules or mass of the lung, active tuberculosis or tuberculosis involving multiple pulmonary lobes, lung atelectasis, interstitial lung diseases or pleura effusion, confirmed by a more than 5-year experienced chest radiologist (n = 83); and 6) subjects with severe heart, liver, and kidney dysfunction (n = 4).

**FIGURE 1:**
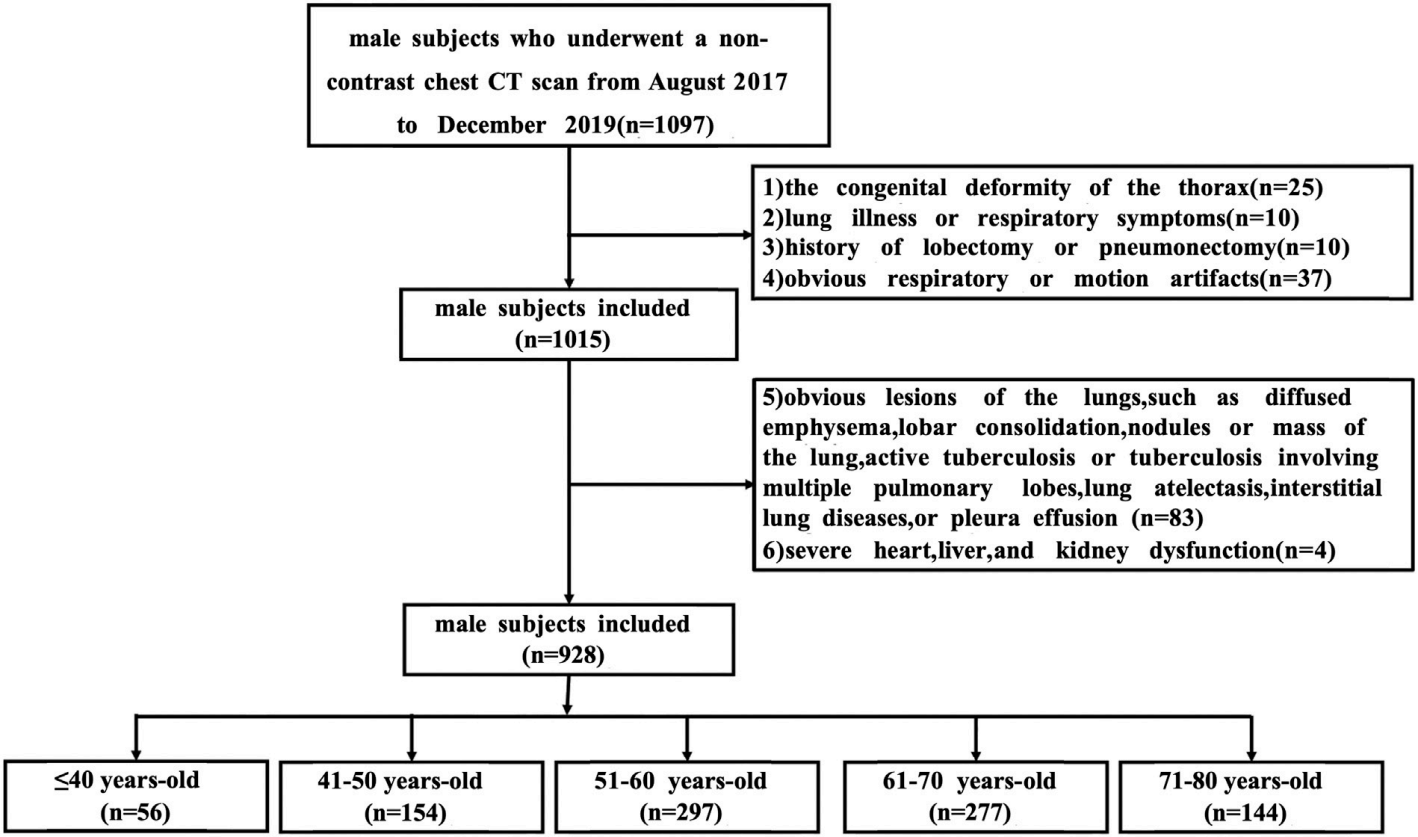
The workflow of subjects. Abbreviations: CT, computed tomography.

Finally, 928 male subjects were included in the analysis. All subjects were grouped by age, with 10 years as an age group, including ≤40 years old (n = 56), 41–50 years old (n = 154), 51–60 years old (n = 297), 61–70 years old (n = 277), 71–80 years old (n = 144). Figure 1 illustrates a flowchart outlining the process for selecting and grouping participants.

### 2.2 Chest CT scan

All CT scans were acquired with the same scanning and reconstruction conditions. CT scans were performed using a 64-MSCT scanner (Gemini TF PET/CT; Philips, Netherlands) from the apex to the base of the lungs at the end-inspiratory phase. An automatic current of 100–300 mAs (based on body weight) and a kilovoltage of 120 were used. Other scanning parameters were held constant: helical acquisition, gantry rotation time of 0.4 s, reconstructed section thickness of 1.25 mm, and reconstructed section interval of 1.25 mm. Images were reconstructed to encompass the entire lung field in a 512 × 512 pixel matrix using the full iterative reconstruction.

### 2.3 Quantitative assessment of the lung structure

Raw data were stored in Digital Imaging and Communications in Medicine format and then transferred to a lung structure analysis workflow to segment the lung field and airways.

**FIGURE 2:**
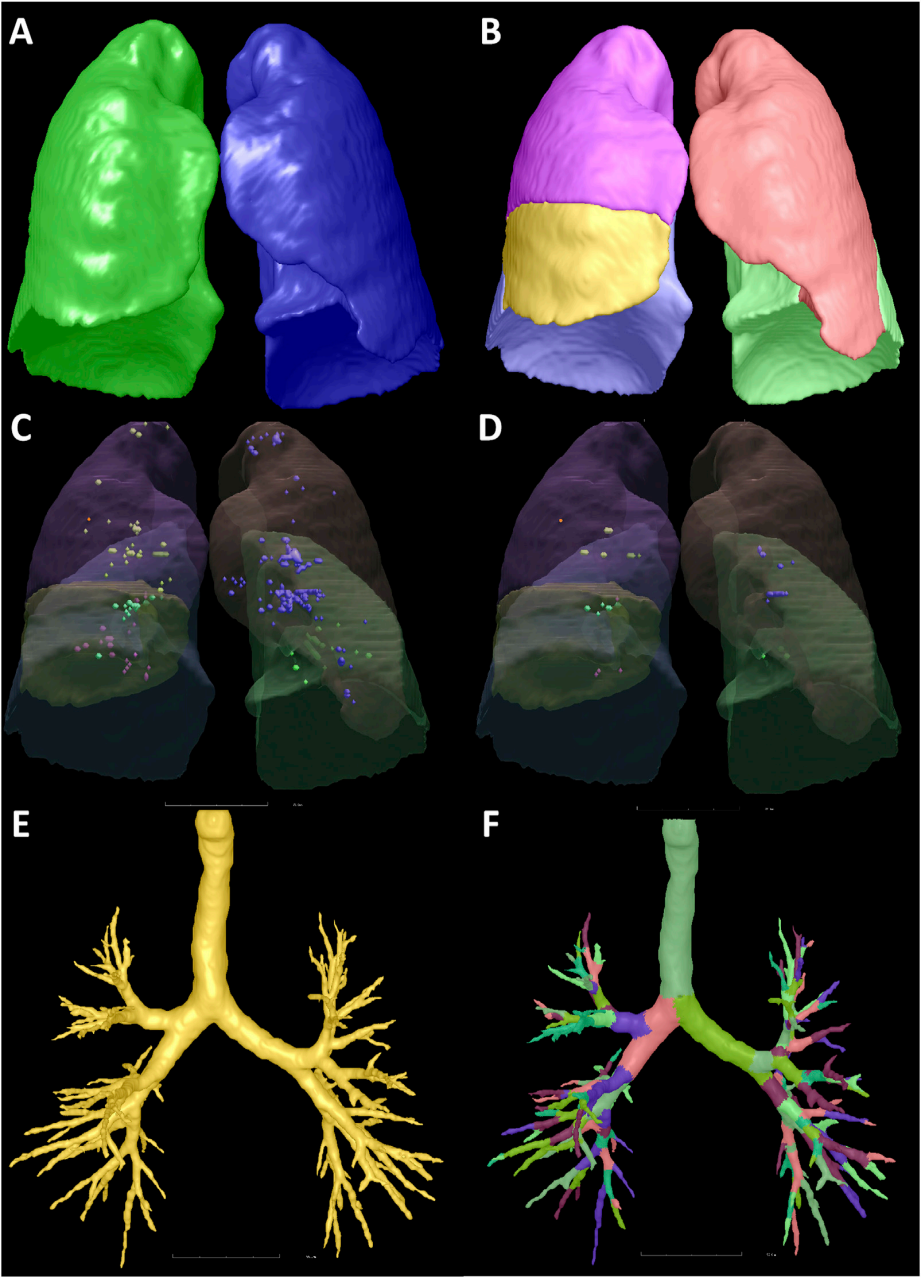
Illustration of the segmentation of the lung, lobes, emphysema, and bronchus. A 53-year-old male with no respiratory symptoms and no apparent abnormalities on the chest CT scan. The bilateral lungs **(A)**, five lobes **(B)**, LAA-910 **(C)**, LAA-950 **(D)**, and the bronchi **(E,F)** were segmented. The LV, LAA-910, LAA-950, MLD, NB, and VB of the total lung were 4562.38 mL, 12.61 mL, 1.73 mL, −770.18 HU, 177 and 77.14 mL, respectively. Abbreviations: CT, computed tomography; LV, lung volume; LAA-910, lower attenuation area than −910 Hounsfield unit; LAA-950, lower attenuation area than −950 Hounsfield unit; MLD, mean lung density; NB, number of bronchi; VB, volume of bronchi.

Automated computerized schemes were used to obtain whole lung field from CT acquisitions. First, the entire lung was segmented using a three-dimensional adaptive border matching algorithm (Figure 2A) (Pu et al., 2009). Pulmonary fissures were detected using computational geometry, and the surfaces of individual lobes were demarcated by representing the pulmonary fissures as implicit surface functions (Figure 2B) (Pu et al., 2011). The results of the computerized segmentation were verified by visual inspection and manually corrected when the computer failed.

Lung volume (LV) of the total lung (TL), right lung (RL), left lung (LL) and five lobes (right upper lobe (RUL), right middle lobe (RML), right lower lobe (RLL), left upper lobe (LUL), and left lower lobe (LLL)) were computed using the segmentation results by counting the voxels circumscribed by the boundaries and multiplying by the volume (mL).

Emphysema was quantified using the percentage of low-attenuation areas below −910 HU (LAA-910) (Figure 2C) and below −950 HU (LAA-950) (Figure 2D). As LAA-910 and LAA-950 were two widely used emphysema metrics (San José Estépar et al., 2025; Yang et al., 2025; Koo et al., 2023). Mean lung density (MLD) is a general indicator of all the components in a given volume of lung, which includes the air, acini, small airways, arteries, veins, capillaries, and epithelial lining fluid. MLD can be quantified by the average density of all the voxels. The ratios of the LAA-910 to LV and LAA-950 to LV were recorded as LAA-910% and LAA-950%, respectively.

The airway tree is automatically recognized and extracted by a geometric algorithm (Pu et al., 2012). The number of bronchi (NB) and the volume of bronchi (VB) were also calculated for TL, RL, LL, and five lobes (Figures 2E,F).

The degree of mismatch between the airway and lung, an indicator of lung function in healthy subjects, is defined as the ratio of airway volume to lung volume (ALR) on a CT scan for TL, RL, LL, and five lobes (Maetani et al., 2023).

### 2.4 Statistical analysis

The following statistical analyses were conducted using IBM SPSS Statistics version 25.0 (IBM Corp., Armonk, NY, United States). Baseline characteristics and quantitative parameters of the lung structures were summarized by age group, using mean ± standard deviation (SD) for normally distributed variables and median (interquartile range) for skewed variables. For variables with a normal distribution, one-way analysis of variance (ANOVA) was used to assess differences among groups; for variables without a normal distribution, the Kruskal-Wallis test was applied. The least significant difference (LSD) for variables with equal variance or Tamhane’s T2 for variables with unequal variance was then used to compare the two groups. A paired t-test or paired samples Wilcoxon Signed-Rank test was used to evaluate the quantitative parameter differences between the right and left lung. The main structural lung changes between one age stage and the previous age stage were identified using binary logistic regression. A two-sided P-value less than 0.05 was considered statistically significant for all statistical analyses.

The following statistical analyses were conducted using the MATLAB (R2023a, The MathWorks, Inc.). A polynomial weighted least squares fitting method incorporating the RLOESS smoothing technique was fitted to visualize the changing trend of quantitative parameters throughout lung aging, where the weighting scheme is adaptively determined through an iterative robust estimation process.

The LungAge model was conducted using R language (version 4.0.5). The correlation analysis was used to initially select correlated parameters. Then, the generalized additive model (GAM), a flexible nonlinear model that extends the generalized linear model by replacing linear predictors with additive functions, was applied to select the most discriminative quantitative parameters. Then, a machine learning model method, eXtreme Gradient Boosting, was used to build a predictive model. Then, mean squared error (MSE), root mean squared error (RMSE), mean absolute error (MAE), and mean absolute percentage error (MAPE) were calculated to evaluate the model, and a 5-fold cross-validation was applied to test its performance by R^2^. Finally, the potential confounders, height, weight, smoking history, and BMI, were analyzed.

## 3 Results

### 3.1 Age-related lung structure alterations of the TL, RL and LL

Totally, 928 males from an annual lung nodule screening cohort aged 25–85 were divided into five subgroups. The height and weight of71–80 years-old were significantly lower than those of the former three groups (*P* < 0.05). The BMI were not significantly different among the five age groups (*P* > 0.05).

For the TL, RL and LL, LV peaked in the group of 51–60 years-old. The LV of the TL and RL of 51–60 years-old was significantly larger than that of ≤40 and 41–50 years-old (*P* < 0.05) (as shown in Table 1).

**TABLE 1:** Basic demographic characteristics of the patients.

Items	≤40 years-old (n = 56)	41–50 years-old (n = 154)	51–60 years-old (n = 297)	61–70 years-old (n = 277)	71–80 years-old (n = 144)	F	*P*
height/cm	174.35 ± 5.41	172.85 ± 4.80^a^	172.88 ± 5.16^a^	171.83 ± 5.16^a^	169.77 ± 6.06^a^	11.501	**0.013**
weight/kg	74.93 ± 11.63	74.40 ± 10.76	75.86 ± 9.65	72.87 ± 8.95	69.70 ± 10.11^a^ ^,^ ^b^ ^,^ ^c^ ^,^ ^d^	13.505	**<0.001**
BMI/kg^e^m^-2^	24.44 ± 2.51	24.98 ± 3.11	24.80 ± 2.66	24.85 ± 2.93	24.40 ± 3.24	0.963	0.613
Smoking (no/yes)	14/42^b^	37/117	115/182^b^	127/150^b^	81/63^b^	41.594^e^	**<0.001**
Smoking intensity (cigarette/day)	15 (11, 21)	21 (11, 21)	21 (11, 21)	21 (11, 21)	11 (7, 21)	2.772^f^	0.597
LV_TL_(mL)	4994.42 ± 1,044.59	5070.02 ± 933.74	5277.16 ± 1,009.35^a^ ^,^ ^b^	5172.71 ± 965.27	5086.35 ± 953.62	2.098	0.063
LV_RL_ (mL)	2,648.94 ± 544.23	2,718.21 ± 481.68	2,841.63 ± 510.89^a^ ^,^ ^b^	2,796.27 ± 502.62^a^ ^,^ ^c^	2,750.40 ± 485.26	2.657	**0.021**
LV_LL_ (mL)	2,345.49 ± 514.74	2,351.81 ± 465.14	2,435.53 ± 516.33	2,376.44 ± 487.49	2,335.95 ± 497.48^c^	1.684	0.133
NB_TL_	160.86 ± 58.64	187.30 ± 64.80	182.50 (146, 238)^a^	214.75 ± 82.03^a^ ^,^ ^b^	200.65 ± 77.41^a^	31.700	**<0.001**
NB_RL_	90.25 ± 36.33	105.56 ± 38.46	104 (81, 133)^a^	111 (86, 145)^a^	105.84 ± 44.50	21.176	**0.001**
NB_LL_	66.27 ± 27.82	74 (58.5, 92.5)	75 (60, 103)^a^	89.68 ± 41.87^a^ ^,^ ^b^	81 (61, 110.5)^a^	28.588	**<0.001**
VB_TL_(mL)	80.91 (71.86, 95.43)	89.67 ± 20.81	93.84 ± 21.52^a^	98.32 ± 23.16^a^ ^,^ ^b^ ^,^ ^c^	99.33 ± 25.56^a^ ^,^ ^b^	48.666	**<0.001**
VB_RL_ (mL)	16.82 ± 5.80	19.16 ± 5.46	19.5 (15.82, 23.28)^a^	20.88 (16.65, 25.64)^a^ ^,^ ^b^	18.76 (15.05, 24.2)	27.830	**<0.001**
VB_LL_ (mL)	15.98 ± 5.17	17.23 (14.32, 21.14)	17.51 (14.43, 21.51)	19.55 ± 6.86^a^ ^,^ ^b^	18.90 ± 6.61^a^	24.691	**<0.001**
MLD_TL_ (HU)	−816.25 (−841.05, −802.63)	−824.43 (−838.48, −803.11)	−825.94 (−841.58, −805.08)	−830.67 (−845.93, −810.90)	−837.15 (−851.11, −821.62)^a^ ^,^ ^b^ ^,^ ^c^	30.692	**<0.001**
MLD_RL_ (HU)	−817.27 (−840.54, −801.11)	−825.46 (−840.38, −806.66)	−826.94 (−842.16, −806.41)	−830.92 (−845.63, −811.54)	−837.27 (−851.87, −822.16)^a^ ^,^ ^b^ ^,^ ^c^	28.244	**<0.001**
MLD_LL_ (HU)	−815.34 (−838.41, −800.13)	−822.07 (−838.24, −799.15)	−823.99 (−840.30, −801.56)	−827.53 (−845.32, −807.00)	−834.68 (−850.46, −813.66)^a^ ^,^ ^b^ ^,^ ^c^	25.401	**<0.001**
LAA-910_TL_ (mL)	311.01 (128.93, 776.96)	382.32 (142.38, 731.64)	469.57 (169.15, 858.56)	554.85 (188.09, 1,140.11)^b^ ^,^ ^c^	751.59 (417.36, 1,365.13)^a^ ^,^ ^b^ ^,^ ^c^	40.681	**<0.001**
LAA-910_RL_ (mL)	131.86 (56.66, 390)	170.49 (76.59, 393.85)	238.20 (91.41, 479.53)	299.34 (109.75, 621.98)^a^ ^,^ ^b^ ^,^ ^c^	388.92 (220.63, 736.52)^a^ ^,^ ^b^ ^,^ ^c^	47.344	**<0.001**
LAA-910_LL_ (mL)	157.8 (65.07, 361.15)	180.42 (67.62, 349.56)	211.39 (76.23, 415.85)	255.54 (88.22, 544.70)^b^	347.20 (167.72, 618.46)^a^ ^,^ ^b^ ^,^ ^c^	32.003	**<0.001**
LAA-950_TL_ (mL)	18.77 (7.72, 37.28)	18.58 (8.45, 35.06)	20.16 (8.79, 40.17)	24.72 (10.08, 59.88)^b^ ^,^ ^c^	41.74 (21.07, 100.11)^a^ ^,^ ^b^ ^,^ ^c^	56.013	**<0.001**
LAA-950_RL_ (mL)	6.41 (2.45, 12.13)	5.99 (3.13, 12.31)	7.78 (3.61, 17.06)	9.6 (4.07, 26.47)^a^ ^,^ ^b^ ^,^ ^c^	18.34 (7.86, 43.95)^a^ ^,^ ^b^ ^,^ ^c^	71.731	**<0.001**
LAA-950_LL_ (mL)	10.92 (5.48, 24.92)	10.92 (5.55, 22.26)	11.96 (5.29, 25.42)	14.75 (6.29, 36.73)^c^	23.96 (11.56, 53.44)^a^ ^,^ ^b^ ^,^ ^c^	40.563	**<0.001**
LAA-910%_TL_	6.73 (2.58, 15.13)	7.22 (3.32, 14.44)	9.06 (3.53, 15.99)	11.07 (4.25, 20.44)^b^	14.94 (8.98, 24.80)^a^ ^,^ ^b^ ^,^ ^c^ ^,^ ^d^	63.688	**<0.001**
LAA-910%_RL_	5.59 (2.51, 13.09)	6.41 (3.12, 13.17)	8.54 (3.35, 16.20)	10.16 (3.92, 19.69)^a^ ^,^ ^b^	14.33 (8.33, 25.67)^a^ ^,^ ^b^ ^,^ ^c^ ^,^ ^d^	69.430	**<0.001**
LAA-910%_LL_	6.95 (3.10, 15.05)	7.81 (3.51, 14.32)	8.94 (3.45, 16.42)	10.62 (4.13, 21.05)^b^	15.56 (8.15, 24.98)^a^ ^,^ ^b^ ^,^ ^c^ ^,^ ^d^	55.544	**<0.001**
LAA-950%_TL_	0.38 (0.18, 0.68)	0.34 (0.19, 0.68)	0.37 (0.18, 0.76)	0.50 (0.22, 1.08)^b^	0.83 (0.41, 1.83)^a^ ^,^ ^b^ ^,^ ^c^ ^,^ ^d^	92.044	**<0.001**
LAA-950%_RL_	0.26 (0.10, 0.41)	0.23 (0.12, 0.42)	0.26 (0.13, 0.61)	0.34 (0.15, 0.92)^b^	0.69 (0.29, 1.60)^a^ ^,^ ^b^ ^,^ ^c^ ^,^ ^d^	109.867	**<0.001**
LAA-950%_LL_	0.49 (0.25, 1.05)	0.47 (0.25, 0.96)	0.50 (0.23, 0.95)	0.63 (0.29, 1.45)	1.04 (0.53, 2.19)^a^ ^,^ ^b^ ^,^ ^c^ ^,^ ^d^	72.666	**<0.001**
ALR_TL_	6.73 (2.58, 15.13)	7.22 (3.32, 14.44)	9.06 (3.53, 15.99)	11.07 (4.25, 20.44)^b^	14.94 (8.98, 24.80)^a^ ^,^ ^b^ ^,^ ^c^ ^,^ ^d^	63.688	**<0.001**
ALR_RL_	5.59 (2.51, 13.09)	6.41 (3.12, 13.17)	8.54 (3.35, 16.20)	10.16 (3.92, 19.69)^a^ ^,^ ^b^	14.33 (8.33, 25.67)^a^ ^,^ ^b^ ^,^ ^c^ ^,^ ^d^	69.430	**<0.001**
ALR_LL_	6.95 (3.10, 15.05)	7.81 (3.51, 14.32)	8.94 (3.45, 16.42)	10.62 (4.13, 21.05)^b^	15.56 (8.15, 24.98)^a^ ^,^ ^b^ ^,^ ^c^ ^,^ ^d^	55.544	**<0.001**

Note: LV, lung volume; NB, number of branches; VB, volume of branches; MLD, mean lung density; LAA, low attenuation area; ALR, airway-to-lung ratio; TL, total lung; RL, right lung; LL, left lung.

^a^ Significantly different from group ≤40 years-old (*P* < 0.05).

^b^ Significantly different from group 41–50 years-old (*P* < 0.05).

^c^ Significantly different from group 51–60 years-old3 (*P* < 0.05).

^d^ Significantly different from group61-70 years-old (*P* < 0.05).

^e^ tested by chi-squared test.

^f^ test by nonparametric method.

The bold values indicate statistically significant differences between groups.

For the TL, RL and LL, NB peaked at 61–70 years-old. The NB in the 51–60 and 61–70 years-old age groups was significantly higher than that in the ≤40 years old group (*P* < 0.05) (as shown in Table 1).

The VB of TL peaked in the group of 71–80 years-old, and VB of RL and LL peaked at 61–70 years-old. For the TL, RL and LL, the VB of 61–70 years-old was significantly higher than that of the ≤40 and 41–50 years-old (*P* < 0.05) (as shown in Table 1).

MLD decreased with age, while LAA-910, LAA-950, LAA-910%, LAA-950%, and ALR all showed an increasing trend with age. MLD was significantly lower in the 71–80 years-old group than that of the ≤40 years-old, 41–50 years-old, and 51–60 years-old groups, while LAA-910%, LAA950%, and ALR of 71–80 years-old were significantly higher than those of the other four age groups (*P* < 0.05) (as shown in Table 1).

In all five age groups, LV, NB, and VB of the RL were larger than those of the LL (*P* < 0.05). MLD, LAA-950, LAA-950%, and ALR of the RL were lower than those of the LL (*P* < 0.05) (as shown in Table 2).

**TABLE 2:** The comparison of the quantitative parameters between theRL and the LL.

Age groups	Items	T or Z	P
≤40 years-old	LV_RL_ - LV_LL_	12.480a	**<0.001**
	NB_RL_ - NB_LL_	6.216	**<0.001**
	VB_RL_ - VB_LL_	3.707	**<0.001**
	MLD_RL_ - MLD_LL_	2.364	**0.018**
	LAA-910_RL_ - LAA-910_LL_	0.624	0.532
	LAA-950_RL_ - LAA-950_LL_	5.911	**<0.001**
	LAA-910%_RL_ - LAA-910%_LL_	3.156	**0.002**
	LAA-950%_RL_ - LAA-950%_LL_	6.298	**<0.001**
	ALR_RL_ - ALR_LL_	5.196	**<0.001**
41–50 years-old	LV_RL_ - LV_LL_	70.279a	**<0.001**
	NB_RL_ - NB_LL_	11.393	**<0.001**
	VB_RL_ - VB_LL_	7.172	**<0.001**
	MLD_RL_ - MLD_LL_	5.399	**<0.001**
	LAA-910_RL_ - LAA-910_LL_	1.209	0.227
	LAA-950_RL_ - LAA-950_LL_	8.622	**<0.001**
	LAA-910%_RL_ - LAA-910%_LL_	3.626	**<0.001**
	LAA-950%_RL_ - LAA-950%_LL_	10.073	**<0.001**
	ALR_RL_ - ALR_LL_	7.333	**<0.001**
51–60 years-old	LV_RL_ - LV_LL_	36.685a	**<0.001**
	NB_RL_ - NB_LL_	14.053	**<0.001**
	VB_RL_ - VB_LL_	10.751	**<0.001**
	MLD_RL_ - MLD_LL_	7.128	**<0.001**
	LAA-910_RL_ - LAA-910_LL_	4.615	**<0.001**
	LAA-950_RL_ - LAA-950_LL_	9.353	**<0.001**
	LAA-910%_RL_ - LAA-910%_LL_	2.948	**0.003**
	LAA-950%_RL_ - LAA-950%_LL_	11.789	**<0.001**
	ALR_RL_ - ALR_LL_	11.429	**<0.001**
61–70 years-old	LV_RL_ - LV_LL_	31.483a	**<0.001**
	NB_RL_ - NB_LL_	13.348	**<0.001**
	VB_RL_ - VB_LL_	10.253	**<0.001**
	MLD_RL_ - MLD_LL_	3.763	**<0.001**
	LAA-910_RL_ - LAA-910_LL_	6.322	**<0.001**
	LAA-950_RL_ - LAA-950_LL_	7.308	**<0.001**
	LAA-910%_RL_ - LAA-910%_LL_	2.626	**0.009**
	LAA-950%_RL_ - LAA-950%_LL_	11.015	**<0.001**
	ALR_RL_ - ALR_LL_	11.053	**<0.001**
71–80 years-old	LV_RL_ - LV_LL_	20.898a	**<0.001**
	NB_RL_ - NB_LL_	8.665	**<0.001**
	VB_RL_ - VB_LL_	4.547	**<0.001**
	MLD_RL_ - MLD_LL_	4.401	**<0.001**
	LAA-910_RL_ - LAA-910_LL_	5.781	**<0.001**
	LAA-950_RL_ - LAA-950_LL_	4.200	**<0.001**
	LAA-910%_RL_ - LAA-910%_LL_	0.227	0.820
	LAA-950%_RL_ - LAA-950%_LL_	6.964	**<0.001**
	ALR_RL_ - ALR_LL_	9.225	**<0.001**

Note: a, paired *t-test*; T and Z were the test statistics of the paired t-test and the paired samples Wilcoxon Signed-Rank test, respectively. LV, lung volume; NB, number of branches; VB, volume of branches; MLD, mean lung density; LAA, low attenuation area; ALR, airway-to-lung ratio; RL, right lung; LL, left lung. The bold values indicate statistically significant differences between groups.

### 3.2 Age-related lung structure alterations of individual lobes

The LV of bilateral upper lobes increased with age, while a decline of LV of bilateral lower lobes was observed since the age group of the sixties. The significance was observed in theRLL between the age groups of 61–70 years-old and 51–60 years-old, as well as between the 71–80 years-old and 51–60 years-old groups (*P* < 0.05) (as shown in Table 3).

**TABLE 3:** The comparisons of quantitative parameters for the individual lobes.

Items	≤40 years-old (n = 56)	41–50 years-old (n = 154)	51–60 years-old (n = 297)	61–70 years-old (n = 277)	71–80 years-old (n = 144)	F	*P*
LV_RUL_ (mL)	1,013.37 ± 274.63	1,020.86 (884.66, 1,201.50)	1,079.5 (919.33, 1,250.26)	1,081.87 (931.29, 1,272.16)	1,115.92 ± 293.00	11.186	**0.048**
LV_RML_ (mL)	211.92 (91.40, 374.15)	188.95 (83.64, 444.58)	276.06 (118.93, 527.35)	333.15 (108.9, 520.34)	287.97 (93.49, 505.31)	12.260	**0.031**
LV_RLL_ (mL)	1,364.35 ± 407.78	1,403.69 ± 410.96	1,419.25 ± 412.35	1,345.35 ± 394.50^a^	1,312.06 ± 380.16^b^ ^,^ ^a^	2.139	0.059
LV_LUL_ (mL)	1,246.19 (1,068.38, 1,397.45)	1,257.74 (1,103.16, 1,382.77)	1,331.02 ± 273.47	1,311.47 ± 249.47	1,301.25 ± 287.23	9.537	0.089
LV_LLL_ (mL)	1,123.61 ± 264.57	1,086.92 ± 275.57	1,104.50 ± 309.27	1,064.95 ± 300.70	933.37 (795.01, 1,148.34)	12.427	**0.029**
NB _RUL_	29 (22.5, 37.5)	33 (24, 43)	32 (25, 42)	35 (26, 47)^c^	33.98 ± 14.29	12.282	**0.031**
NB _RML_	13 (11, 17)	17 (11, 22)	17 (13, 25)^c^	19 (13, 26)^c^	17 (11.5, 25)	26.335	**<0.001**
NB _RLL_	43.59 ± 19.20	51.74 ± 22.34	51 (38, 65)	52 (38, 71)^c^	51.60 ± 25.31	15.747	**0.008**
NB _LUL_	29 (20, 35)	32 (25, 43)	32 (25, 46)	37 (27, 49)^c^	36 (26, 49.5)^c^	22.807	**<0.001**
NB _LLL_	35.27 ± 16.36	42 (31, 51)	42 (31, 57)^c^	49.34 ± 25.80^c^	45.5 (30, 60)^c^	24.496	**<0.001**
VB_RUL_ (mL)	3.51 ± 1.37	4.02 ± 1.46	3.89 (3.13, 5.02)	4.35 (3.36, 5.45)^c^	4.17 ± 1.50^c^	20.883	**0.001**
VB_RML_ (mL)	1.83 (1.27, 2.75)	2.38 ± 1.02	2.45 (1.78, 3.27)^c^	2.72 (1.86, 3.54)^c^	2.39 ± 1.17	25.669	**<0.001**
VB_RLL_ (mL)	4.83 ± 2.49	5.43 (3.72, 7.11)	5.66 (4.03, 7.62)	6.08 (4.31, 8.84)^c^	5.29 (3.64, 7.79)	19.717	**0.001**
VB_LUL_ (mL)	4.12 ± 1.68	4.50 (3.46, 5.51)	4.60 (3.45, 5.82)	5.17 ± 2.19^c^ ^,^ ^b^	4.75 (3.43, 6.31)	21.525	**0.001**
VB_LLL_ (mL)	5.46 ± 2.51	6.32 ± 2.63	6.10 (4.57, 8.28)	7.32 ± 3.65^c^	6.30 (4.61, 8.82)^c^	22.983	**<0.001**
MLD_RUL_ (HU)	−830.86 (−851.56, −813.01)	−834.46 (−848.26, −818.81)	−835.27 (−849.58, −818.90)	−839.48 (−854.02, −821.44)	−845.15 (−858.95, −830.46)^c^ ^,^ ^b^ ^,^ ^a^	33.469	**<0.001**
MLD_RML_ (HU)	−823.25 (−838.95, −793.41)	−826.95 (−842.82, −802.37)	−832.63 (−852.19, −807.55)	−836.10 (−855.35, −812.69)^c^ ^,^ ^b^	−843.29 (−862.50, −817.49)^c^ ^,^ ^b^ ^,^ ^a^	40.832	**<0.001**
MLD_RLL_ (HU)	−810.38 (−831.34, −789.61)	−816.18 (−834.83, −793.31)	−815.77 (−834.16, −786.49)	−818.93 (−838.51, −795.74)	−826.24 (−839.20, −805.19)^a^	15.601	**0.008**
MLD_LUL_ (HU)	−832.35 (−853.44, −818.11)	−836.55 (−850.41, −820.54)	−838.74 (−852.46, −820.78)	−842.01 (−856.74, −824.00)	−848.86 (−862.44, −830.39)^c^ ^,^ ^b^ ^,^ ^a^	27.955	**<0.001**
MLD_LLL_ (HU)	−800.35 (−825.18, −775.74)	−804.18 (−827.16, −773.78)	−803.11 (−825.13, −774.67)	−808.89 (−831.53, −781.47)	−817.46 (−836.36, −789.11)^b^ ^,^ ^a^	14.698	**0.012**
LAA-910_RUL_ (mL)	62.99 (17.78, 162.46)	62.55 (27.51, 169.50)	96.37 (33.58, 212.40)	114.82 (40.22, 269.78)^b^ ^,^ ^a^	164.81 (87.64, 347.51)^c^ ^,^ ^b^ ^,^ ^a^	41.477	**<0.001**
LAA-910_RML_ (mL)	4.49 (0.21, 25.22)	5.51 (0.42, 40.59)	17.11 (1.41, 85.33)^b^	25.64 (1.59, 110.62)^c^ ^,^ ^b^	37.64 (5.24, 141.35)^c^ ^,^ ^b^ ^,^ ^a^	44.218	**<0.001**
LAA-910_RLL_ (mL)	59.58 (22.21, 152.86)	68.85 (16.76, 166.94)	80.57 (16.53, 189.62)	91.98 (30.40, 227.76)	142.04 (63.67, 241.95)^c^ ^,^ ^b^ ^,^ ^a^ ^,^ ^d^	28.371	**<0.001**
LAA-910_LUL_ (mL)	124.58 (53.27, 273.63)	141.00 (57.22, 261.79)	165.96 (61.41, 314.75)	199.64 (70.88, 405.62)^b^	246.61 (133.16, 466.68)^c^ ^,^ ^b^ ^,^ ^a^	31.183	**<0.001**
LAA-910_LLL_ (mL)	35.20 (12.34, 94.00)	34.93 (8.29, 108.87)	37.87 (10.48, 103.36)	51.06 (16.26, 143.20)	82.64 (28.34, 174.67)^c^ ^,^ ^b^ ^,^ ^a^	28.591	**<0.001**
LAA-950_RUL_ (mL)	2.41 (0.7, 5.47)	1.73 (0.87, 4.14)	2.12 (0.99, 5.19)	2.69 (1.15, 7.37)^b^ ^,^ ^a^	4.97 (1.91, 12.21)^c^ ^,^ ^b^ ^,^ ^a^	48.353	**<0.001**
LAA-950_RML_ (mL)	0.13 (0.01, 0.94)	0.16 (0.01, 1.10)	0.51 (0.05, 2.49)^c^ ^,^ ^b^	0.83 (0.07, 4.92)^c^ ^,^ ^b^	1.62 (0.15, 7.26)^c^ ^,^ ^b^ ^,^ ^a^	52.901	**<0.001**
LAA-950_RLL_ (mL)	2.81 (0.8, 6.33)	2.51 (0.86, 7.81)	2.79 (0.96, 6.61)	3.74 (1.41, 10.29)	7.69 (2.78, 16.71)^c^ ^,^ ^b^ ^,^ ^a^ ^,^ ^d^	50.062	**<0.001**
LAA-950_LUL_ (mL)	8.5 (4.25, 16.25)	7.13 (3.82, 14.72)	8.05 (3.54, 16.52)	9.45 (4.14, 25.55)	16.08 (7.24, 35.91)^c^ ^,^ ^b^ ^,^ ^a^ ^,^ ^d^	39.766	**<0.001**
LAA-950_LLL_ (mL)	2.79 (0.84, 8.50)	2.45 (1.11, 8.44)	2.93 (1.06, 7.26)	4.27 (1.73, 11.12)^a^	6.98 (2.5, 16.49)^c^ ^,^ ^b^ ^,^ ^a^	34.686	**<0.001**
LAA-910%_RUL_	6.46 (2.36, 17.24)	6.26 (3.04, 15.49)	8.90 (3.40, 18.18)	10.85 (3.86, 22.59)^b^	15.77 (8.77, 28.40)^c^ ^,^ ^b^ ^,^ ^a^ ^,^ ^d^	62.369	**<0.001**
LAA-910%_RML_	2.22 (0.19, 9.10)	3.34 (0.62, 14.28)	7.74 (0.96, 19.83)^c^	9.48 (1.84, 23.79)^c^ ^,^ ^b^	17.09 (4.92, 32.58)^c^ ^,^ ^b^ ^,^ ^a^ ^,^ ^d^	62.370	**<0.001**
LAA-910%_RLL_	4.45 (1.81, 9.00)	4.73 (1.46, 11.90)	5.87 (1.35, 12.46)	6.57 (2.46, 16.45)	11.47 (5.74, 18.69)^c^ ^,^ ^b^ ^,^ ^a^ ^,^ ^d^	57.584	**<0.001**
LAA-910%_LUL_	9.81 (4.81, 20.77)	10.99 (5.13, 19.95)	12.23 (5.18, 22.92)	14.68 (5.98, 26.71)	19.63 (11.01, 32.96)^c^ ^,^ ^b^ ^,^ ^a^ ^,^ ^d^	48.533	**<0.001**
LAA-910%_LLL_	3.06 (1.29, 7.61)	2.99 (0.95, 9.00)	3.68 (1.06, 8.69)	5.09 (1.71, 12.42)^b^	8.36 (3.60, 16.83)^c^ ^,^ ^b^ ^,^ ^a^ ^,^ ^d^	49.103	**<0.001**
LAA-950%_RUL_	0.24 (0.08, 0.44)	0.18 (0.09, 0.36)	0.18 (0.10, 0.45)	0.24 (0.12, 0.60)^b^	0.51 (0.18, 1.11)^c^ ^,^ ^b^ ^,^ ^a^ ^,^ ^d^	82.022	**<0.001**
LAA-950%_RML_	0.07 (0.00, 0.24)	0.07 (0.02, 0.37)	0.21 (0.03, 0.66)^c^ ^,^ ^b^	0.28 (0.05, 0.98)^c^ ^,^ ^b^	0.58 (0.12, 1.72)^c^ ^,^ ^b^ ^,^ ^a^ ^,^ ^d^	78.313	**<0.001**
LAA-950%_RLL_	0.20 (0.07, 0.39)	0.18 (0.07, 0.46)	0.19 (0.08, 0.46)	0.28 (0.11, 0.72)^b^ ^,^ ^a^	0.57 (0.24, 1.24)^c^ ^,^ ^b^ ^,^ ^a^ ^,^ ^d^	96.073	**<0.001**
LAA-950%_LUL_	0.68 (0.37, 1.19)	0.56 (0.32, 1.15)	0.65 (0.28, 1.24)	0.72 (0.32, 1.73)	1.23 (0.60, 2.67)^c^ ^,^ ^b^ ^,^ ^a^ ^,^ ^d^	65.103	**<0.001**
LAA-950%_LLL_	0.26 (0.09, 0.68)	0.24 (0.11, 0.72)	0.28 (0.11, 0.61)	0.42 (0.17, 1.02)^b^ ^,^ ^a^	0.71 (0.29, 1.43)^c^ ^,^ ^b^ ^,^ ^a^ ^,^ ^d^	62.141	**<0.001**
ALR_RUL_	6.46 (2.36, 17.24)	6.26 (3.04, 15.49)	8.90 (3.40, 18.18)	10.85 (3.86, 22.59)^b^	15.77 (8.77, 28.40)^c^ ^,^ ^b^ ^,^ ^a^ ^,^ ^d^	62.369	**<0.001**
ALR_RML_	2.22 (0.19, 9.10)	3.34 (0.62, 14.28)	7.74 (0.96, 19.83)^c^	9.48 (1.84, 23.79)^c^ ^,^ ^b^	17.09 (4.92, 32.58)^c^ ^,^ ^b^ ^,^ ^a^ ^,^ ^d^	62.370	**<0.001**
ALR_RLL_	4.45 (1.81, 9.00)	4.73 (1.46, 11.90)	5.87 (1.35, 12.46)	6.57 (2.46, 16.45)	11.47 (5.74, 18.69)^c^ ^,^ ^b^ ^,^ ^a^ ^,^ ^d^	57.584	**<0.001**
ALR_LUL_	9.81 (4.81, 20.77)	10.99 (5.13, 19.95)	12.23 (5.18, 22.92)	14.68 (5.98, 26.71)	19.63 (11.01, 32.96)^c^ ^,^ ^b^ ^,^ ^a^ ^,^ ^d^	48.533	**<0.001**
ALR_LLL_	3.06 (1.29, 7.61)	2.99 (0.95, 9.00)	3.68 (1.06, 8.69)	5.09 (1.71, 12.42)^b^	8.36 (3.60, 16.83)^c^ ^,^ ^b^ ^,^ ^a^ ^,^ ^d^	49.103	**<0.001**

Note: LV, lung volume; NB, number of branches; VB, volume of branches; MLD, mean lung density; LAA, low attenuation area; ALR, airway-to-lung ratio; RUL, right upper lobe; RML, right middle; RLL, right lower lobe; LUL, left upper lobe; LLL, left lower lobe.

^c^ Significantly different from group ≤40 years-old (*P* < 0.05).

^b^ Significantly different from group 41–50 years-old (*P* < 0.05).

^a^ Significantly different from group 51–60 years-old (*P* < 0.05).

^d^ Significantly different from group 61–70 years-old (*P* < 0.05).

The bold values indicate statistically significant differences between groups.

VB and NB in all five lobes of the lungs increased gradually with age and peaked in the group of 61–70 years. The NB and VB in all five lobes of the 61–70 years-old were greater than those of the ≤40 years-old significantly (*P* < 0.05) (as shown in Table 3).

MLD of the bilateral upper lobes and the RML was diminished with age, and the significance was observed between the group of 71–80 years-old and the groups of ≤40 years-old, 41–50 years-old, and 51–60 years-old (*P* < 0.05). The MLD of the bilateral lower lobes decreased, and a significant difference was observed between the groups of 71–80 years-old and 51–60 years-old (*P* < 0.05) (as shown in Table 3).

LAA-910, LAA-950, LAA-910%, LAA-950%, and ALR increased gradually with age in all five lobes (all *P* < 0.05). The LAA-910%, LAA-950%, and ALR of the 71–80 years-old in all five lobes were higher than those of the other four groups (*P* < 0.05) (as shown in Table 3).

**FIGURE 3:**
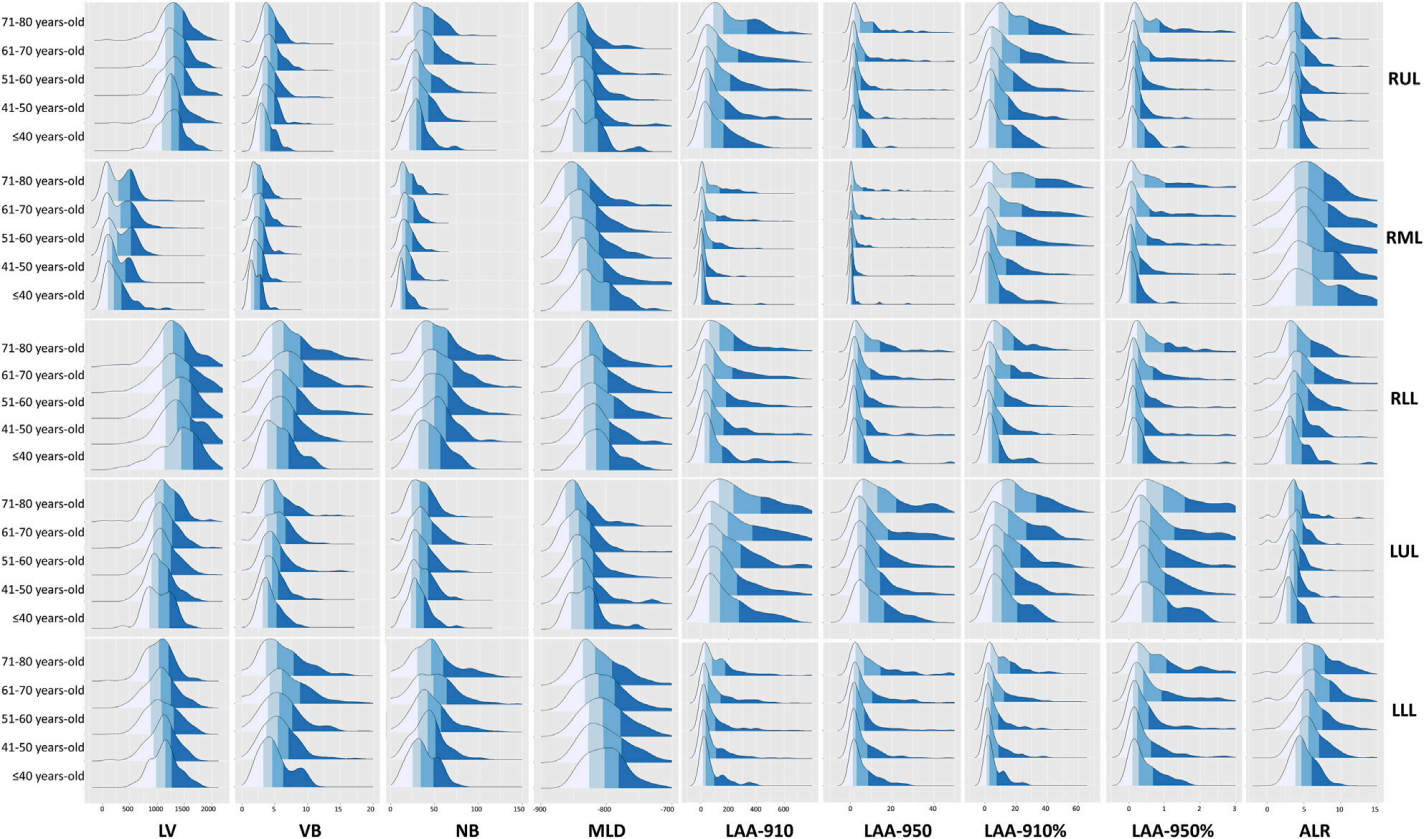
The ridge plot of the quantitative parameters of each lobe. Abbreviations: LV, lung volume; LAA-910, lower attenuation area than −910 Hounsfield unit; LAA-950, lower attenuation area than −950 Hounsfield unit; MLD, mean lung density; NB, number of bronchi; VB, volume of bronchi; RUL, right upper lobe; RML, right middle; LUL, left upper lobe; LLL, left lower lobe.

The ridge plot was used for illustration of age-related lung changes at the lobe level, see as Figure 3.

### 3.3 Curve fitting of the quantitative parameters

**FIGURE 4:**
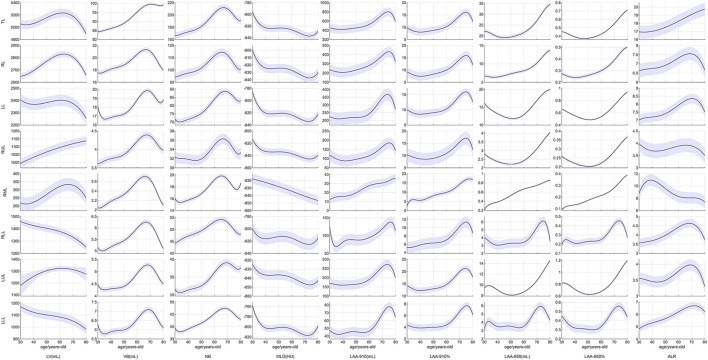
Curve fitting of the quantitative parameters. Abbreviations: LV, lung volume; LAA-910, lower attenuation area than −910 Hounsfield unit; LAA-950, lower attenuation area than −950 Hounsfield unit; MLD, mean lung density; NB, number of bronchi; VB, volume of bronchi; RUL, right upper lobe; RML, right middle; LUL, left upper lobe; LLL, left lower lobe.

The fitted curves helped to reveal the trajectories of the quantitative parameters (see Figure 4). LV of TL, RL, LL and LUL peaked at about 60 years-old. LV of RUL exhibited a monotonic increase without decline, whereas LV of bilateral lower lobes demonstrated progressive reduction. Both VB and NB reached their peak values between 61-70 years of age.

The MLD showed a rapid decline from the age of 30–40 years-old, remained stable or slightly increased during 40–50 years-old, then began to decline again after 50 years-old. A final turning point occurred at 70 years-old, after which MLD exhibited a slight upward trend. However, the MLD of the RML consistently demonstrated a decreasing trend throughout.

LAA-910 and LAA-910% exhibited a mild decrease during ages 30–40, followed by a sustained increase until 60 years, after which a rapid increase and a downward trend were observed. LAA-950 and LAA-950% of TL decreased during ages 30–50, and then progressively increased with aging, and a similar trend of LAA-950 and LAA-950% can be seen in LL, RUL and LUL. However, the LAA-950 and LAA-950% of RLL and LLL displayed a steeper increase began at 60 years old, and finished at the 71–80 years old.

### 3.4 Main changes between two adjacent age groups

We compared the differences in the quantitative parameters between the two adjacent age groups. Using binary logistic regression, we identified the most significant changes between one age stage and the previous age stage, as shown in Table 4. The increase in ALR of LLL was the most significant characteristic between the age group ≤40 and 41–50 years old [OR = 1.315, 95% CI (1.094, 1.580), *P* = 0.003]. The most important lung structural changes from the age of 51–60 years-old to the age of 61–70 years-old pointed to LAA-910 of LLL and LAA-910% of LLL. The most important lung structural changes from the age of 61–70 years-old to the age of 71–80 years-old were the emphysema indexes.

**TABLE 4:** The most significant quantitative parameters between two adjacent age groups.

Comparisons	Items	*b*	*P*	OR	95% CI	
					Lower limit	Upper limit
≤40 years-old vs*.* 41–50 years-old	ALR_LLL_	0.274	**0.003**	1.315	1.094	1.580
41–50 years-old vs*.* 51–60 years-old	LV_RUL_	0.001	**0.017**	1.001	1.000	1.002
	LV_RML_	0.002	**<0.001**	1.002	1.001	1.003
	Constant	−0.985	**0.044**	0.374		
51–60 years-old vs*.* 61–70 years-old	ALR_TL_	0.127	**<0.001**	1.136	1.076	1.199
	LAA-910_LLL_	−0.011	**0.001**	0.989	0.982	0.995
	LAA-910%_LLL_	0.203	**<0.001**	1.225	1.111	1.351
	Constant	−3.078	**<0.001**	0.046		
61–70 years-old vs*.* 71–80 years-old	LAA-950_LL_	−0.053	**<0.001**	0.948	0.922	0.974
	LAA-950%_LL_	1.109	**0.017**	3.030	1.220	7.526
	LAA-950%_LUL_	0.588	**0.019**	1.800	1.102	2.938
	Constant	−1.210	**<0.001**	0.298		

Note: LV, lung volume; LAA, low-attenuation area; RUL, right upper lobe; RML, right middle; LUL, left upper lobe; LLL, left lower lobe; *b*, the coefficient of the selected index; OR, odds ratio; CI, confidence interval. The bold values indicate statistically significant differences between groups.

### 3.5 The LungAge score

**FIGURE 5:**
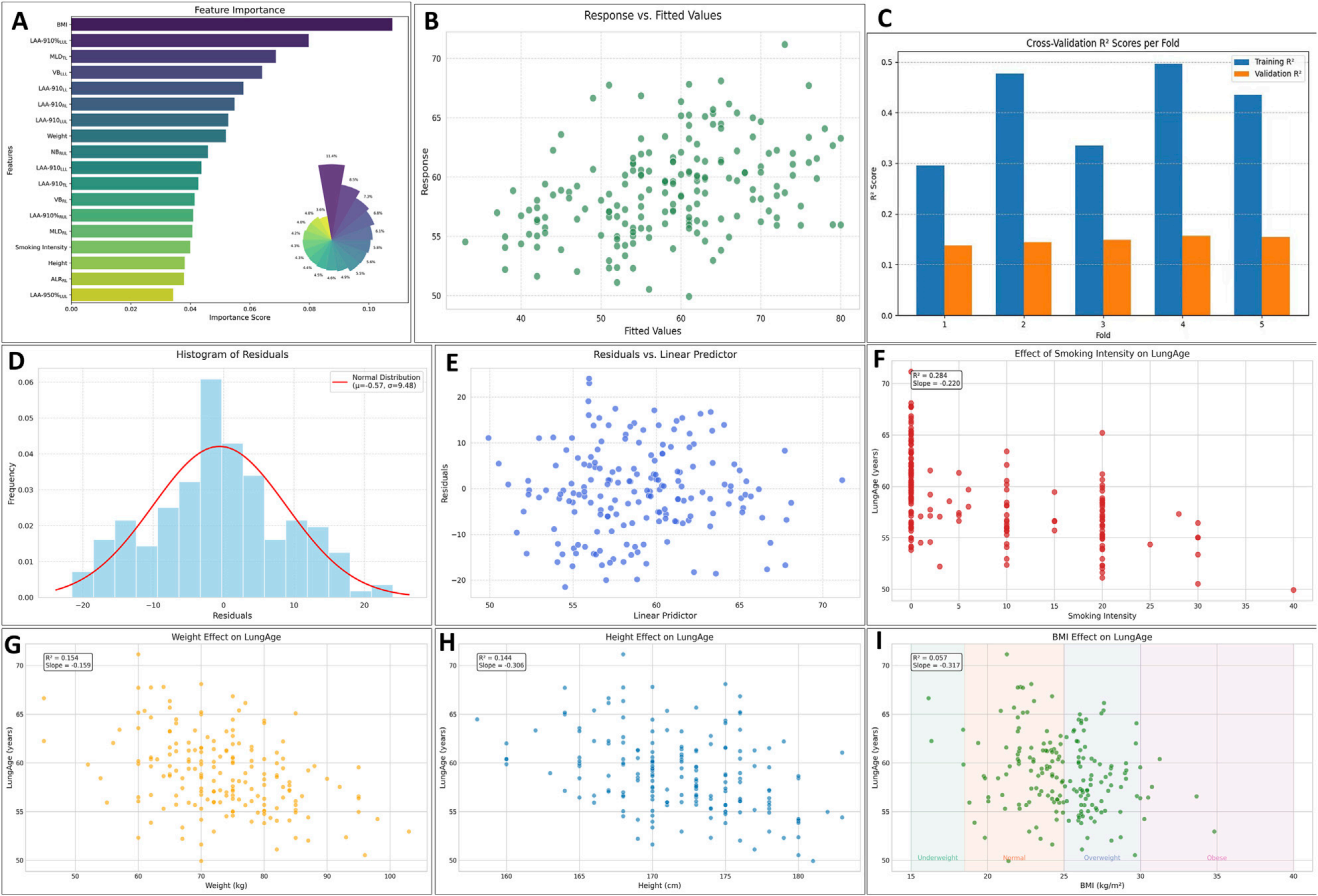
The evaluation of the lungAge Score. The selected parameters and their importance were shown in **(A)**. The fitted value vs. the response value was shown in **(B)**. In the 5-fold cross-validation, the average R^2^ in the training group was 0.408, and the average R^2^ in the testing group was 0.148, as shown in **(C)**. The residual plot showed normal distribution, as shown in **(D, E)**. The potential confounders, smoking intensity, height, weight, and BMI, were analyzed to determine their effect on the LungAge score, as shown in **(F-I)**. Abbreviations: LV, lung volume; LAA-910, lower attenuation area than −910 Hounsfield unit; LAA-950, lower attenuation area than −950 Hounsfield unit; MLD, mean lung density; NB, number of bronchi; VB, volume of bronchi; RUL, right upper lobe; RML, right middle; LUL, left upper lobe; LLL, left lower lobe.

Spearman correlation was firstly used to identify the related parameters during lung aging (see Supplementary Table). The selected parameters in the GAM and their importance were shown in Figure 5A. The fitted value vs. the response value were shown in Figure 5B. The MSE, RMSE, MAE, and MAPE were 89.789, 9.475, 7.478, and 13.76%. In the 5-fold cross-validation, the average R^2^ in the training group was 0.408, the average R^2^ in the testing group was 0.148, see as Figure 5C. The residual plot showed normal distribution, see as Figures 5D,E. Finally, the potential confounders, smoking intensity, height, weight, and BMI, were analyzed to determine their effect on the LungAge score, as shown in Figures 5F–I.

## 4 Discussions

By the time we are in our second or third decade, biological aging and chronological aging do not proceed in step (Bowdish, 2019). We hypothesized that this inconsistency can be measured by some predictors that are related to the lungs. This study analyzed age-related structural changes of the aging lung in a relatively large dataset. The patterns were analyzed not only at the level of the entire lung and bilateral lungs but also at the level of individual lobes.

These quantitative parameters (LV, NB, VB, MLD, LAA-910, LAA-950, LAA-910%, LAA-950% and ALR) reflect age-related lung changes from different angles. LV and MLD are both comprehensive measurements of the composition and proportion of pulmonary vessels, gas, and lung tissues. VB can be seen as part of the anatomical dead space, which reflects, in part, the residual volume in the lung function test. The increase in NB also attributed to the increase in residual volume in the lungs with age, and can be viewed as a biomarker of small airways (Verleden et al., 2020). LAA-910, LAA-910%, LAA-950 and LAA-950% were mainly pointed to the emphysematous changes. ALR was proved to be a biomarker of impaired pulmonary function in COPD (Maetani et al., 2023; Tanabe et al., 2019). All the quantitative parameters were used to assess the effect of aging, and several novel findings were identified.

First, lung aging is not a linear process in quantitative analysis. LV peaked at about 60 years old, and VB and NB reached their peak values between 61 and 70 years of age. The MLD declined from 30 to 40 years of age with minor fluctuations during the fifth decade (40–50 years), and turned a slight upward trend at 70 years. LAA-910 and LAA-910% exhibited a mild decrease during ages 30–40, followed by a sustained increase until 60 years, after which a downward trend was observed. These data suggest compensatory structural changes in the lungs to maintain ventilation function at the age of 50–60 years old. However, as age exceeds 70 years-old, this compensatory mechanism may gradually fail. Data from the lung function showed that both the forced vital capacity (FVC) and forced expiratory volume for one second (FEV1) peaked at 20–30 years old, and then declined (Erelund et al., 2023; Sahebi et al., 2022). A recent study found that aging is not a linear process (Shen et al., 2024), and a notable decrease in oxygen carrier activity around age 60.

Second, lung aging shows different change patterns between the upper lobes and the lower lobes. For the LV, RUL increased without a decline trend, but the bilateral lower lobes declined throughout the ages. The pattern of the LV of bilateral lobes is consistent with the lung function parameters; this can be explained by the bilateral lower lobes being more correlated with lung function. The paired inspiratory-expiratory chest CT quantitative results from Wu et al. (2021) showed that the volumetric change of the lower lobes was larger than the upper lobes. They also showed that the CT quantitative indexes derived from LLL and RUL gave a strong correlation with TLC and FVC. However, the CT quantitative indexes derived from the right lung (RUL and RML) were associated with Li et al. (2024). Also discovered that the LLL plays the largest role in ventilation among the five lobes, reminding us that the LLL is more sensitive to lung function decline than the other four lobes. For the LAA-950 and LAA-950%, a turning point was observed at approximately 60 years old in the bilateral lower lobes, but not in the bilateral upper lobes. These data suggest that the bilateral upper lobes were predominantly affected by emphysema (Li et al., 2023), while the bilateral lower lobes were predominantly affected by interstitial disease (Choi et al., 2022).

Third, the most obvious changes between one age stage and the previous age stage were identified using binary logistic regression. A previous study found that emphysema increased with age, ranging from 0.01% at age 40–50 years to 0.4% at age 70–80 years (Martinez et al., 2017). Our findings are consistent with previous studies. This accelerated lung aging, considered as a normal age-related senile emphysema, may be part of COPD pathogenesis (Yoon et al., 2019). Studying lung structure in normal subjects provides a basis for detecting abnormalities at an early stage (Cheng et al., 2019; Jacob et al., 2019; Ritchie et al., 2024). We have also developed a LungAge score, which can be used to assess the structural lung aging features.

However, this study has several limitations. First, despite the relatively large size of the dataset, the sample size is not equally distributed in each age group. The sample size of the age ≤40 years old and >70 years old was relatively small. Thus, the present findings are subject to confirmation in males aged ≤40 years and >70 years. It is recommended that the sample size be further expanded in future studies. Second, this is a cross-sectional study; tracking individuals longitudinally over longer periods will be required to observe these trajectories at an individual level. Third, all the subjects were from a single center, which may limit the applicability of the results in the external sample. Fourth, the quantitative parameters used in the study are limited when calculating a more precise LungAge score. More sensitive biomarkers need to be defined or designed to help us understand the changes of the aging lung. Fifth, a previous study from our team has confirmed that the low-dose CT scan (compared to the standard-dose) and the traditional reconstruction technique (filtered back projection) will lead to higher LAA and higher noise (Huang et al., 2020). In clinical practice, most health management centers or hospitals prefer a low-dose CT scan for lung nodular screening; therefore, advanced image reconstruction should be considered to decrease noise and achieve optimal LAA values (Ferri et al., 2022; Bak et al., 2020). Sixth, the lack of lung function data limits our consideration to older individuals who maintain their functionality despite illnesses and diseases. As we were trying to display the age-related changes of lung structure with regard to the “healthy aging” population. Finally, there is a lack of data on pulmonary function. We would collect pulmonary function data to better interpret the trajectories of the quantitative parameters.

## 5 Conclusion

In summary, our findings demonstrated that lung aging is not a linear process, with peak ages for LV and VB occurring at 51–60 and 61–70 years-old, respectively, while the progression of emphysema becomes particularly pronounced after 70 years of age. Age-related lung structural alterations in the upper and lower lobes exhibit significant heterogeneity, with the upper lobes predominantly demonstrating volume expansion accompanied by marked emphysema progression, whereas the bilateral lower lobes primarily show volume reduction with interstitial fibrotic proliferation after 60 years-old. This study identifies the parameters and lobes exhibiting the most significant changes between adjacent age groups, and we provide a computational formula, LungAge Score, for the assessment of the structural lung aging features.

## Data Availability

The raw data supporting the conclusions of this article will be made available by the authors, without undue reservation.
