# Supplementary material for: Age-related lung structure changes by quantitative assessment: a cross-sectional study in a Chinese male cohort
Source: Front Aging. 2025 Oct 23;6:1624233. doi: 10.3389/fragi.2025.1624233 (PMC12588894; doi:10.3389/fragi.2025.1624233)
Supplement: Supplementary file 1 [file Table1.docx]

**Supplementary table Correlations of the all the quantitative parameters with age**

| Items | *r* | *p* | Items | *r* | *p* |
| --- | --- | --- | --- | --- | --- |
| LV_TL_ | 0.009 | 0.784 | LV_RML_ | 0.084 | **0.010** |
| LAA-910_TL_ | 0.217 | **<0.001** | LAA-910_RML_ | 0.223 | **<0.001** |
| LAA-950_TL_ | 0.246 | **<0.001** | LAA-950_RML_ | 0.243 | **<0.001** |
| LAA-910%_TL_ | 0.246 | **<0.001** | LAA-910%_RML_ | 0.266 | **<0.001** |
| LAA-950%_TL_ | 0.269 | **<0.001** | LAA-950%_RML_ | 0.288 | **<0.001** |
| MLD_TL_ | -0.181 | **<0.001** | MLD_RML_ | -0.217 | **<0.001** |
| NB_TL_ | 0.144 | **<0.001** | NB_RML_ | 0.103 | **0.002** |
| VB_TL_ | 0.218 | **<0.001** | VB_RML_ | 0.087 | **0.008** |
| ALR_TL_ | 0.253 | **<0.001** | ALR_RML_ | -0.075 | **0.023** |
| LV_RL_ | 0.035 | 0.285 | LV_RLL_ | -0.100 | **0.002** |
| LAA-910_RL_ | 0.235 | **<0.001** | LAA-910_RLL_ | 0.171 | **<0.001** |
| LAA-950_RL_ | 0.287 | **<0.001** | LAA-950_RLL_ | 0.226 | **<0.001** |
| LAA-910%_RL_ | 0.258 | **<0.001** | LAA-910%_RLL_ | 0.223 | **<0.001** |
| LAA-950%_RL_ | 0.304 | **<0.001** | LAA-950%_RLL_ | 0.275 | **<0.001** |
| MLD_RL_ | -0.174 | **<0.001** | MLD_RLL_ | -0.122 | **<0.001** |
| NB_RL_ | 0.088 | **0.007** | NB_RLL_ | 0.064 | 0.052 |
| VB_RL_ | 0.114 | **<0.001** | VB_RLL_ | 0.096 | **0.003** |
| ALR_RL_ | 0.113 | **0.001** | ALR_RLL_ | 0.147 | **<0.001** |
| LV_LL_ | -0.017 | 0.608 | LV_LUL_ | 0.070 | **0.033** |
| LAA-910_LL_ | 0.192 | **<0.001** | LAA-910_LUL_ | 0.191 | **<0.001** |
| LAA-950_LL_ | 0.203 | **<0.001** | LAA-950_LUL_ | 0.195 | **<0.001** |
| LAA-910%_LL_ | 0.227 | **<0.001** | LAA-910%_LUL_ | 0.210 | **<0.001** |
| LAA-950%_LL_ | 0.233 | **<0.001** | LAA-950%_LUL_ | 0.207 | **<0.001** |
| MLD_LL_ | -0.161 | **<0.001** | MLD_LUL_ | -0.167 | **<0.001** |
| NB_LL_ | 0.152 | **<0.001** | NB_LUL_ | 0.151 | **<0.001** |
| VB_LL_ | 0.146 | **<0.001** | VB_LUL_ | 0.137 | **<0.001** |
| ALR_LL_ | 0.184 | **<0.001** | ALR_LUL_ | 0.114 | **0.001** |
| LV_RUL_ | 0.104 | **0.001** | LV_LLL_ | -0.096 | **0.003** |
| LAA-910_RUL_ | 0.220 | **<0.001** | LAA-910_LLL_ | 0.177 | **<0.001** |
| LAA-950_RUL_ | 0.236 | **<0.001** | LAA-950_LLL_ | 0.192 | **<0.001** |
| LAA-910%_RUL_ | 0.232 | **<0.001** | LAA-910%_LLL_ | 0.221 | **<0.001** |
| LAA-950%_RUL_ | 0.241 | **<0.001** | LAA-950%_LLL_ | 0.235 | **<0.001** |
| MLD_RUL_ | -0.173 | **<0.001** | MLD_LLL_ | -0.124 | **<0.001** |
| NB_RUL_ | 0.075 | **0.023** | NB_LLL_ | 0.127 | **<0.001** |
| VB_RUL_ | 0.117 | **<0.001** | VB_LLL_ | 0.128 | **<0.001** |
| ALR_RUL_ | 0.038 | 0.243 | ALR_LLL_ | 0.201 | **<0.001** |

Note: LV, lung volume; NB, number of branches; VB, volume of branches; MLD, mean lung density; LAA, low attenuation area; ALR, airway-to-lung ratio; TL, total lung; RL, right lung; LL, left lung; RUL, right upper lobe; RML, right middle; RLL, right lower lobe; LUL, left upper lobe; LLL, left lower lobe.
